# Dosimetric comparison of organs at risk using different contouring guidelines for definition of the clinical target volume in anal cancer

**DOI:** 10.1007/s00066-020-01587-y

**Published:** 2020-02-03

**Authors:** Hendrik Dapper, Markus Oechsner, Stefan Münch, Christian Diehl, Jan C. Peeken, Kai Borm, Stephanie E. Combs

**Affiliations:** 1 Department of Radiation Oncology, Klinikum rechts der Isar, TU München, Ismaninger Str. 22, 81675 Munich, Germany; 2 Partner Site Munich, Deutsches Konsortium für Translationale Krebsforschung (DKTK), Munich, Germany; 3 Institute for Radiation Medicine (IRM), Helmholtz Zentrum München, Ingolstädter Landstr. 1, Neuherberg, 85764 Germany

**Keywords:** Anal cancer, Contouring guidelines, Organs at risk, Dose distribution, Inguinal lymph nodes

## Abstract

**Background:**

There are different contouring guidelines for definition of the clinical target volume (CTV) for intensity-modulated radiation therapy (IMRT) of anal cancer (AC). We conducted a planning comparison study to evaluate and compare the dose to relevant organs at risk (OARs) while using different CTV definitions.

**Methods:**

Twelve patients with a primary diagnosis of anal cancer, who were treated with primary chemoradiation (CRT), were selected. We generated four guideline-specific CTVs and subsequently planned target volumes (PTVs) on the planning CT scan of each patient. An IMRT plan for volumetric arc therapy (VMAT) was set up for each PTV. Dose parameters of the planned target volume (PTV) and OARs were evaluated and compared, too.

**Results:**

The mean volume of the four PTVs ranged from 2138 cc to 2433 cc. The target volumes contoured by the authors based on the recommendations of each group were similar in the pelvis, while they differed significantly in the inguinal region. There were no significant differences between the four target volumes with regard to the dose parameters of the cranially located OARs. Conversely, some dose parameters concerning the genitals and the skin varied significantly among the different guidelines.

**Conclusion:**

The four contouring guidelines differ significantly concerning the inguinal region. In order to avoid inguinal recurrence and to protect relevant OARs, further investigations are needed to generate uniform standards for definition of the elective clinical target volume in the inguinal region.

## Background

Anal cancer (AC) is a relatively rare malignant tumor of the lower gastrointestinal tract. Chemoradiation (CRT) has been established as an organ-preserving standard approach in non-metastatic disease and is associated with good overall survival rates [1, 2]. So far, the most relevant prognostic factors correlating with survival endpoints are locoregional lymph node (LN) involvement, primary tumor size >5 cm, and pathological complete response [3]. Currently, three international recommendations for intensity-modulated radiation therapy (IMRT) exist for definition of the clinical target volume (CTV) of AC. These were published by the Radiation Therapy Oncology Group (RTOG), the Australasian Gastro-Intestinal Trial Group (AGITG), and the authors and collaborative groups of the British National Guidance (BNG) [4–6]. The AGITG rightly pointed out that there is currently not sufficient evidence regarding the definition of the inguinal lymphatic drainage [6]. The inguinal region is affected primarily or metachronously in up to 40% of all AC patients [7–9]. In a recent PET imaging-based analysis of patterns of LN involvement in primary AC, we (the lead institution) were able to demonstrate that the three guidelines differ in their hypothetical effectiveness to cover microscopic LN metastases. Based on these results, we worked out a recommendation for CTV delineation of the inguinal region [10]. Achieving the best possible locoregional control is an important objective in the treatment of AC. However, typical complications such as dermatological, genitourinary, and gastrointestinal side effects, and others like vaginal stenosis, hip osteoarthritis, or sexual dysfunction play a decisive role and should always be considered during treatment planning [11–15].

In order to be able to estimate the efficiency regarding locoregional control rates and potential side effects of the different guidelines, we conducted a comparative study on the various CTVs and determined the dose to OARs.

## Methods

We selected 12 patients with the primary diagnosis of squamous cell AC who were treated with CRT at our institution between 2012 and 2018. In all patients, we retrospectively generated the three different CTVs of the established guidelines and a fourth CTV using our own guidelines (LI) on the original planning CT scan with a slice thickness of 3 mm (all prone position). All guidelines are very similar regarding their recommendations for delineation of the elective LN regions in the pelvis (internal/external iliac, pre-sacral, internal obturatoric). There are, however, significant differences regarding the mesorectal and ischiorectal fossa and especially the inguinal region. For the essential contents of the guidelines, see Table 1. With regard to uncertainties leaving room for interpretation of the different definitions of the target volume, formulations such as “should be contoured as a compartment,” we tried to refer as much as possible to the respective atlases published. The planning target volume of the inguinal region (PTV^ing^) was created by a 6-mm outer margin of the respective CTV. The planning target volume of the pelvic region which included the primary tumor region (PTV^pelvic^) was conceived by increasing the CTV by 10 mm in all directions. The PTV was always limited to the surface of the body. Afterwards, mostly based on the pelvic normal tissue atlas of RTOG, the vulva, scrotum, femoral heads, small bowel, rectum, sigmoid colon, and the urinary bladder were defined [16]. The skin was determined as the volume that represents the first 3 mm of the body surface into the body. We also outlined the genitalia analogously to the genitalia contouring guidelines of Brooks et al. [17].

**Table 1:** Overview of the three major guidelines and the TUM guideline for elective CTV definition for IMRT of primary anal cancer

Elective CTV	RTOG	AGITG	BNG	TUM
**Cranial**	**All: **bifurcation of the common iliac artery/level of the recto-sigmoid junction *RTOG: or 2* *cm above the most cranial aspect of a macroscopic tumor* *BNG: if N0 mesorectal: the lower 50* *mm of the mesorectum*			
**Pelvis**	**All: **inclusion of internal and external iliac, presacral nodes, para-rectal, mesorectal nodes *AGITG: inclusion of the ischiorectal fossa*			
**Caudal** **(inguinal LN)**	*Radial:*	*Radial:*	*Radial:*	*Radial:*
	“As a compartment with any identified nodes”	“As a compartment”, anterior 20 mm and medial 10–20 mm of femoral vessels	“As a compartment”, anterior 5 mm from skin and medial the spermatic cord	2 cm of femoral vessels. 1 cm of great saphenous vein. 3 cm at superomedial and superolateral superficial nodes
	*Caudal:*	*Caudal:*	*Caudal:*	*Caudal:*
	2 cm caudal to the saphenous/femoral junction	Lower edge of the ischial tuberosities	Lesser trochanter	Anal verge, high risk: inclusion of ano-inguinal lymphatic drainage

*CTV* clinical target volume; *LN* lymph nodes; *RTOG* Radiation Therapy Oncology Group; *AGITG* Australasian Gastro-Intestinal Trial Group; *BNG* British National Guidance; *TUM* Technical University Munich (suggestions from retrospective analysis of inguinal patterns of LN involvement); *AILD* ano-inguinal lymphatic drainage

Subsequently, we created a main and a boost plan for the four different distinct PTVs in each individual for volumetric modulated arc therapy (VMAT) intended to be irradiated on a Varian Clinac® DHX linear accelerator (Varian Medical Systems, Palo Alto, CA, USA). The primary tumor region (PTR) and the pelvic LNs were supposed to receive a total dose of 50.4 Gy and the inguinal LNs were supposed to receive a total dose of 36 Gy (single dose 1.8 Gy). For the main plan, the dose prescription was 36 Gy (1.8 Gy single dose) to PTV1 (PTV^pelvic^ + PTV^ing^), which included the PTR and the elective pelvic and inguinal LNs. 14.4 Gy (1.8 Gy single dose) was prescribed to PTV2 (PTV^pelvic^) including the PTR and the elective pelvic LNs, disregarding the inguinal LNs for the sequential boost. The dose constraints for OARs based on Quantitative Analyses of Normal Tissue Effects in the Clinic (QUANTEC) [21]. For VMAT, we regularly used three arcs in the main plan (PTV1) and two arcs for the boost plan (PTV2; 6 or 15 MV). The dose was prescribed to the median of the PTV (ICRU83) [18]. The software used for structure definition and dose comparison was Eclipse Treatment Planning System 13.0 (Varian Medical Systems, Palo Alto, CA, USA).

A Friedman test using SPSS 25.0 (SPSS Inc, Chicago, IL, USA) was applied to identify significant differences between the four plans with regard to all dose parameters of the PTV and the OAR. A post hoc analysis was performed to find out whether any significant differences between the individual plans of RTOG, AGITG, BNG, and LI could be identified. A *p*-value <0.05 was considered to indicate statistical significance.

## Results

### Patients’ characteristics

In accordance with the gender-specific incidence, we chose eight female and four male patients. The median age at diagnosis was 57 years (range: 41–78). Two patients were staged T1, five T2, four T3, and one patient T4. Seven patients initially showed positive LNs. This ultimately resulted in two patients with UICC stage I, three patients with stage II, and seven with stage III disease. Median BMI was 28 kg/m^2^ (range: 17–41).

### PTV volumes and plan value

**Fig. 1:**
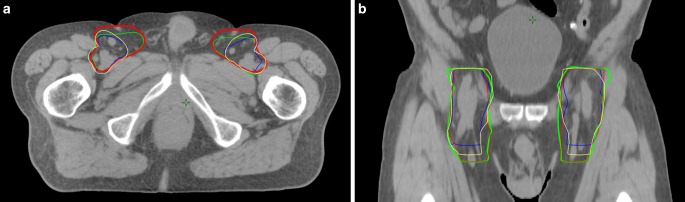
Differences in inguinal clinical target volumes using four different contouring guidelines for intensity-modulated radiotherapy of elective target volumes in primary treatment of anal cancer (AC) (**a** axial, **b** coronal). *Blue* Radiation Therapy Oncology Group, *yellow* Australasian Gastrointestinal Trials Group, *green* Leading Institute, *red* British National Guidance

The mean volumes of the PTVs of the RTOG, AGITG, BNG, and LI groups amounted to 2138 cc, 2407 cc, 2419 cc, and 2433 cc, respectively. In accordance with the distinct contouring guidelines, all CTVs had the same cranial border (the bifurcation of the common iliac artery plus 1 cm). The caudal and radial margins at the inguinale site, however, differed (Table 1*, *Fig. 1). All volumes were significantly larger (≈10%) compared to the volume of the RTOG, whereas no significant differences between the three PTV volumes of the AGITG, BNG, and LI groups could be identified. Taking only the PTV volumes of the inguinal region into consideration, the results of the four groups differed greatly. All inguinal PTV volumes were significantly higher compared to RTOG (<0.001). With 660 cc, the inguinal PTV of the BNG was almost twice as high as the PTV of the RTOG (363 cc). The PTV of the LI, however, was similar to that of the BNG (651 cc). The PTV of the AGITG was 500 cc and thus significantly smaller than the PTV of the BNG (*p* = 0.016).

The dose coverage of all PTVs was accurately performed, which resulted in a mean V95% of 100% and a mean median dose of 50.0 Gy for all PTV^sum^. The mean maximal dose (D2%) came to 52.4 Gy (104%) and the mean minimal dose (D98%) was between 36.2 and 39.8 Gy for all PTV^sum^. The mean V95% of the PTV^ing^ was at least 98% after the PTVs had been defined in accordance with the instructions of the four different guidelines.

### Dose distribution to organs at risk

We evaluated various relevant relative and absolute dose parameters of the rectum, sigmoid colon, small bowel loops, femoral heads, urinary bladder, genitalia, and skin. The results are summarized in Table 2 and 3.

**Table 2:** Absolute dose parameters of organs at risk for different cranial PTVs

Structure	Parameter	RTOG	AGITG	BNG	TUM
		Gy			
*Rectum*	Dmean	50.4	50.2	50.2	50.3
	D98%	48.9	48.9	49.0	49.0
	D2%	52.0	52.1	52.0	52.0
*Sigmoid colon*	Dmean	45.7	45.7	45.8	45.7
	D98%	27.8	28.0	27.9	27.6
	D2%	50.3	50.3	50.4	50.3
*Femoral head*	Dmean	30.0	30.2	30.7	30.0
	D98%	22.9	23.1	23.0	22.5
	D2%	41.3	41.0	41.6	41.2
*Urinary bladder*	Dmean	29.9	30.1	30.2	30.1
	D2%	11.5	11.5	11.9	11.6
	D50%	31.2	31.4	31.5	31.4
	D98%	50.3	50.2	50.4	50.4
*Genitalia*	Dmean	20.5	21.0	**22.0**	22.6
	D98%	5.5	5.9	**6.4**	**6.5**
	D2%	43.8	45.0	44.8	44.7
*Testis (n* *=4)*	Dmean	6.4	7.1	8.0	10.2
	D98%	2.1	2.4	**2.6**	3.0
	D50%	5.3	5.5	**6.4**	**7.9**
	D2%	16.8	20.5	22.1	25.1
*Vulva (n* *=8)*	Dmean	16.2	16.5	17.1	18.2
	D98%	5.3	6.3	6.8	6.6
	D50%	13.6	13.6	14.4	16.6
	D2%	35.1	36.2	35.6	35.8
*Small bowel loops*	20 cc	48.8	48.7	48.8	48.9
	65 cc	45.0	44.9	45.0	44.9
	150 cc	38.8	38.7	38.5	38.4
	200 cc	35.6	35.5	35.4	35.2

*Bold values* statistically significant difference to RTOG

**Table 3:** Relative dose parameters of organs at risk for different cranial PTVs

Structure	Parameter	RTOG	AGITG	BNG	TUM
–	–	**Volume (%)**			
*Rectum*	V45 Gy	100	100	100	100
	V50 Gy	66	65	63	65
*Sigmoid Colon*	V20 Gy	98	98	98	98
	V30 Gy	91	92	91	91
	V40 Gy	85	85	84	85
	V50 Gy	8	7	8	7
*Urinary bladder*	V10 Gy	89	89	90	87
	V20 Gy	65	68	67	66
	V30 Gy	43	43	44	44
	V40 Gy	32	32	32	32
	V50 Gy	7	7	8	8
*Femoral head*	V20 Gy	100	100	100	100
	V30 Gy	44	46	50	45
	V40 Gy	5	4	6	5
*Genitalia*	V10 Gy	78	**81**^a^	**84**	83
	V20 Gy	43	44	**48**	50
	V30 Gy	19	19	22	27
	V40 Gy	12	12	12	15
	V50 Gy	2	2	3	2
*Testis*	V3	17	20	**23**	24
*Vulva*	V30	12	12	12	17
*–*	–	**Volume (cc)**			
*Small bowel loops*	V10 Gy	527	528	527	526
	V20 Gy	438	435	436	437
	V30 Gy	302	297	300	296
	V40 Gy	193	192	194	198
	V50 Gy	32	32	33	32
*Skin*	V10 Gy	358	**388**	**396**	**398**
	V20 Gy	80	**96**	**108**	**107**
	V30 Gy	16	**22**	**29**	**28**
	V35 Gy	7	9	**11**	**11**
	V50 Gy	1	1	1	1

*Bold values* statistically significant difference to RTOG

^a^BNG significant to AGITG

There was no significant difference in dose distribution in the cranially located OARs. The rectum was almost completely included in the different PTVs in all patients (D98% >48.8 Gy). Since the cranial margins of the individual PTVs were identical, there were no significant differences between the dose parameters of the small bowel loops. The mean dose to 65 cc and 200 cc was about 45 and 35 Gy, respectively, for all groups. The relative and absolute doses to the femoral heads and the urinary bladder did not show any significant differences between the four target volumes, either. The Dmean was about 30 Gy for both structures. The mean V40 Gy of the urinary bladder did not reach more than 40% and the mean V30 Gy of the femoral heads was 50% or less.

The dose parameters to the genitalia and the skin, unlike those to the pelvic and abdominal risk structures, showed significant differences. The mean Dmean to the genitalia of both sexes was about 20 Gy in all plans. However, there was a significant difference between BNG (22 Gy) and RTOG (20.5 Gy; *p* = 0.016). The D98% of the BNG (6.4 Gy) and the LI (6.5 Gy) was significantly higher than the D98% of the RTOG (5.3 Gy; *p* = 0.001 and *p* = 0.016, respectively). Despite minor absolute differences, the V10 Gy of the BNG (84%) was significantly higher than those of the RTOG (78%, *p* = 0.016) and the AGITG (81%, *p* = 0.043). The same applies to the V20 Gy. While it was 48% with the BNG, it was 43% with the RTOG (*p* = 0.016). Concerning the vulva (eight patients), the dose parameters did not differ greatly.

The skin turned out to be the organ showing the greatest differences between the dose parameters of the four target volumes. All in all, the results in the BNG and LI groups were the highest and comparable to each other, while the RTOG results were the lowest. The V10–V20 Gy values were significantly lower in the RTOG (358 cc and 80 cc) compared to the three other guidelines (388–398 cc and 96–107 cc). So were V30 Gy and V35 Gy compared to the BNG and the LI (*p* < 0.004). Low doses fluctuated less than higher doses. In the BNG group, for example, the V10 Gy was only about 10% higher (398 cc) than the V10 Gy in the RTOG (358 cc), whereas the clinically more relevant V30 Gy was almost twice as high in the BNG group as in the RTOG (29 cc vs. 16 cc, *p* = 0.000).

## Discussion

We retrospectively evaluated the dose to OAR in twelve patients by using three established international contouring guidelines and one guidance created by the LI for the definition of the elective target volume for IMRT of primary anal cancer. According to the guidelines of the National Comprehensive Cancer Network (NCCN, 2018), IMRT is preferred over 3D conformal RT. The anus and the perineum as well as the pelvic and inguinal LNs should be included in the target volume [19]. Although current contouring atlases refer to IMRT, the common constraints are often related to 3D conformal irradiation [20].

### Pelvic region:

The recommendations of the various guidelines concerning the radial margins of the CTV for the pelvis differ only marginally (Table 1). Especially the cranial margins are identical, since all internal and external iliac nodes up to the bifurcation of the common iliac vessels should be included in the CTV. In our study, the dose parameters were slightly higher than the constraints of 30 Gy (200 cc) and 35 Gy (150 cc), respectively, recommended by RTOG 0529 [12]. One explanation may be the fact that, depending on tumor stage, the dose prescription of elective LNs was 42 Gy or 45 Gy in RTOG 0529. In contrast to this, we prescribed 50.4 Gy. Furthermore, the V30 Gy and V40 Gy have been identified as significant dose parameters regarding acute gastrointestinal toxicity using IMRT in anal cancer patients. DeFoe et al. suggest a V30 Gy ≤ 310 cc and V40 Gy ≤ 70 cc to avoid ≥ grade 3 toxicity (CTCAE), while Devisetty et al. observed a correlation between V30 Gy > 450 cc with 2B and higher (RTOG) gastrointestinal toxicity [21, 22]. All four groups came up with the V30 Gy ≤ 310 cc (about 300 cc) while the V40 Gy was about 200 cc. A further argument for the rather high dose prescription for the pelvic lymph drainage is in our opinion the fact that in four patients, the PTV was slightly above the bifurcation of the iliac artery due to extensive locoregional LN involvement. In addition, due to anatomical conditions, the urinary bladder is not greatly influenced by the different PTV^ing^.

### Inguinal region:

The total dose of the elective inguinal target volume used in large prospective trials ranges from 30.6 Gy (ACT II) to 45 Gy (RTOG 98–11), which means an enormous divergence [1, 2]. As there is still uncertainty about the optimal elective dose to the inguinal region, we opted for a compromise and chose a total prescription dose of 36 Gy. While the target volumes and the dose to OARs hardly differed in the pelvis, there were large differences in the inguinal region between the guidelines. The BNG and LI groups recommend inclusion of the superomedial LNs (spermatic cord in men). This means that the CTV below the groin extends much further medially than the CTV suggested by the RTOG and the AGITG. The BNG group suggest that the CTV should reach up to 5 mm to the skin. The most obvious differences between the recommendations of the individual groups can be found with regard to the caudal margin of the elective CTV (Table 1; Fig. 1). Here, the height of bony structures (ischial tuberosities/AGITG, trochanter/BNG) as well as soft tissue structures (2 cm caudal to the saphenous femoral junction/RTOG; height of anal verge/LI) is recommended. In a previous study, we were able to show that the anatomical relationships between these soft tissue structures and bony structures differ significantly between patients [10]. Therefore, evidence-based recommendations for target volume definition of the inguinal region in anal cancer are needed.

**Fig. 2:**
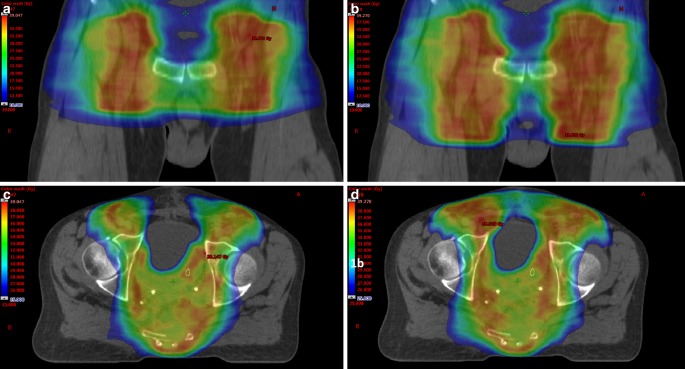
Differences in inguinal dose distribution using the clinical target volume (CTV) definition of the Radiation Therapy Oncology Group (RTOG; **a** and **c**) and the British National Guidance (BNG; **b** and **d**). Axial color wash: 25 Gy (*dark blue*)–39 Gy (*dark red*). Transversal color wash: 10 Gy (*dark blue*) –39 Gy (*dark red*). CTV of BNG expands more caudally and medially and leads to a significantly greater dose to the skin and the genitalia

The fundamentally dreaded side effects of radiotherapy to the groin are lymphatic edema caused by inguinal fibrosis. The pronounced medial and caudal extensions of the target volume suggested by the BNG and LI groups lead in part to significantly higher dose rates to the genitals (Fig. 2; Tables 2, 3). Regarding RTOG 0630 (sarcoma), the median dose to the testis should not extend 3 Gy [23]. In each group, the median dose to the testis was higher than 5 Gy. Even if the absolute dose to the testicles is relatively low, we recommend considering special protection of the genitals. The D50% of the vulva was below 17 Gy in each group (RTOG 0529 constraints: 30 Gy). The risk of developing genital lymphedema is very low if a relatively low dose of 36 Gy is prescribed. The University of Florida presented long-term toxicity in 164 patients after elective radiation (>70% of the patients with ≥45 Gy) of the groin in pelvic cancers. Only three of them developed mild genital edema [24].

The greatest relative and absolute differences concerning the dose parameters between the different CTVs related to the skin. We authenticated a correlation between the skin dose and the inguinal PTV volumes. The caudal margin of the CTV is especially responsible for an increased volume. This is due to the fact that the inferior aspect of the minor tuberculum (BNG) and the level of the anal verge (LI) are usually situated lower than the ischial tuberosities (AGITG) or 2 cm below the femoral saphenous junction (RTOG; Fig. 1). The inguinal target volume was almost twice as high in the BNG and LI groups compared to RTOG (V30 Gy and V35 Gy, respectively). This aspect, however, seems to be of minor clinical relevance since the absolute dose radiation to the groin (36 Gy) is relatively low. Lee et al. identified 13 of 164 patients (8%) with inguinal fibrosis after elective radiation (>70% of the patients with ≥45 Gy) of the groin. None of these cases was severe or correlated with a decrease in quality of life [24].

**Fig. 3:**
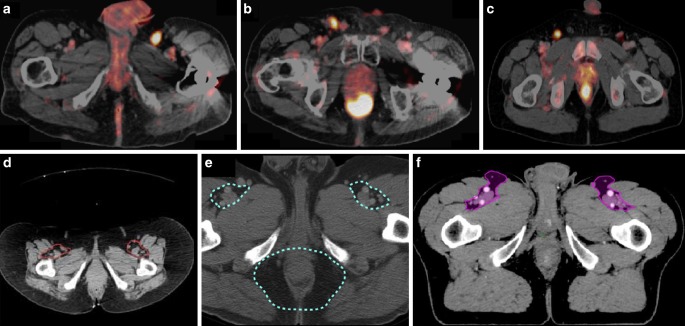
PET-positive superomedial superficial inguinal lymph nodes (LNs) (**a**–**c**) in patients with primary diagnosis of anal cancer. The LNs were possibly not properly covered by the elective clinical target volume (CTV) recommendations of the Radiation Therapy Oncology Group (RTOG, *red outline*; **d**) and the Australasian Gastrointestinal Trials Group (AGITG, *turquoise outline*; **e**), but completely included in the CTV of the British National Guidance (BNG, *purple outline*; **f**)

To make clear recommendations for contouring of the inguinal region, results of studies dealing with the site of locoregional failure after IMRT of AC patients should be taken into consideration. Tomasoa et al. presented patterns of recurrence in 106 patients treated with simultaneously integrated boost (SIB) IMRT. After a median follow-up of 15 months, about 20% of the patients had locoregional relapse. Only two LN recurrences occurred at a pelvic site, while at least four patients had inguinal recurrences (4%, 6 LN) [25]. Furthermore, potential inguinal misses such the ones on superficial superomedial LNs could be identified in a PET imaging-based pattern of failure analysis in the context of established contouring guidelines ([26]; Fig. 3). Finally, the risk of relevant side effects due to slightly larger inguinal target volumes (BNG, LI) seems to be low, since both the absolute size differences and the prescribed dose are manageable, while inguinal recurrence is still relevant.

### Limitations:

All in all, we evaluated a small number of patients. In daily practice, physicians perform a risk-adapted contouring of individual cases with individual anatomy, which will certainly differ from the different guidelines. Moreover, it is difficult to standardize terms like “the inguinal region should be contoured as a compartment with any identified nodes.” Therefore, the CTVs we created can only be seen as an approximation of an elective “standard” CTV of the respective guidelines. The data, however, provide valuable information on the possibilities of contouring in anal cancer.

## Conclusion

The four contouring guidelines differ significantly concerning the inguinal region. In order to avoid inguinal recurrence and to protect relevant OARs, further investigations are needed to generate uniform standards for the definition of the elective clinical target volume in the inguinal region.

## Abbreviations

AC: Anal cancer
AGITG: Australasian Gastrointestinal Trial Group
BNG: British National Guidance
CRT: Chemoradiation
CT: Computed tomography
CTCAE: Common Terminology Criteria for Adverse Events
CTV: Clinical target volume
Gy: Gray
IMRT: Intensity-modulated radiotherapy
LI: Lead institution
LN: Lymph node(s)
MRI: Magnetic resonance imaging
PET: Positron-emission tomography
PTR: Primary tumor region
PTV: Planned target volume
RT: Radiation therapy
RTOG: Radiation Therapy Oncology Group
VMAT: Volumetric arc therapy
